# Perceived barriers and rewards to sexual consent communication: A qualitative analysis

**DOI:** 10.1177/02654075221080744

**Published:** 2022-03-12

**Authors:** Jessica Edwards, Uzma S Rehman, E Sandra Byers

**Affiliations:** 1 Department of Psychology, 8430 University of Waterloo, Waterloo, ON, Canada; 2 Department of Psychology, 3427 University of New Brunswick, Fredericton, ON, Canada

**Keywords:** sexual consent, sexual communication, affirmative consent

## Abstract

Increasingly, affirmative consent – direct, unambiguous and voluntary agreement to engage in sexual activity (Craig & McKinley, 2015) – is the standard being adopted by educational institutions in North America (Bennett, 2016). Yet, studies show that most individuals continue to communicate consent through nonresistance (Jozkowski et al., 2014a). Given this discrepancy, it is critical to understand what factors prevent individuals from engaging in affirmative consent. Furthermore, a better understanding of the perceived rewards of consent communication could incentivize the adoption of affirmative consent. To understand the range of perceived barriers and rewards, we conducted an online, qualitative study where 231 participants answered two open-ended questions. We used inductive content analysis to categorize participants’ perceptions of sexual consent barriers and rewards into four general content areas: (1) Communication Quality, (2) Relational and Emotional Experiences, (3) Sexual Quality and (4) Safety and Coercion. These perceived rewards and barriers were examined through the lens of the Information-Motivation-Behavioural Skills Model. Participants viewed consent communication not only as a means of ensuring safety but also as a way to enhance relational and sexual quality. However, they also perceived barriers in all three of these domains as well as barriers to ensuring that sexual consent communication is fluid and easily understood. These findings provide important avenues for future research investigating how individuals reconcile perceived rewards and costs of affirmative consent communication. We also suggest ways to enhance sexual education by discussing potential rewards and validating the normative nature of fears and anxieties around affirmative consent.

Affirmative sexual consent has been defined as a clear, unambiguous and voluntary agreement to engage in sexual activity (Craig & McKinley, 2015) and is viewed as an important mechanism to reduce instances of sexual coercion and assault (Shumlich & Fisher, 2019). It is increasingly being implemented as the standard for consensual sexual behaviour in administrative regulations, legal stipulations and programs designed to promote sexual health literacy (Shumlich & Fisher, 2019). In 2014, California and New York passed legislation that required all public campuses within each state to adopt affirmative consent as the standard for consensual sexual activity (Jozkowski, 2015). By 2016, an estimated 1500 US colleges and universities have made affirmative consent the standard for consensual sexual behaviour (Bennett, 2016).

Studies show that there is a gap between standards and practice, such that the majority of individuals continue to communicate consent primarily through non-resistance rather than using direct and explicit forms of communication (Jozkowski et al., 2014b). However, reliance on silence or non-resistance to indicate consent places greater responsibility on sexual assault victims rather than perpetrators (Pineau, 1989) and is legally insufficient as a form of consent in jurisdictions such as Canada (Criminal Code of Canada, 1985, s. 273.1(1)), New York and California (Jozkowski, 2015). Given the importance of this topic, there has been an increase in conceptual (Harris, 2018; Jozkowski, 2015; Muehlenhard et al., 2016; Shumlich & Fisher, 2019; Willis & Jozkowski, 2018) and empirical (Curtis & Burnett, 2017; Shumlich & Fisher, 2020) research investigating different aspects of sexual consent communication, including a focus on barriers to affirmative consent communication (Jozkowski, 2015; Wignall, 2020; Willis & Jozkowski, 2018).

In contrast, less attention has been paid to perceived rewards of affirmative consent communication (see Marcantonio et al., 2020, Shumlich & Fisher, 2020 and Piemonte et al., 2020, as recent exceptions that have investigated perceived benefits). The concept of sexual consent as potentially *rewarding* is a novel direction that could incentivize people to adopt affirmative consent practices. Although a sex-positive, reward-focused view of consent is becoming more common in educational discourse and in research (Beres, 2007; 2020), without empirical evidence supporting the idea that affirmative consent can be experienced as rewarding, such discourse can be criticized as conjecture. Also, a better understanding of the types of personal and relational benefits that some individuals receive from affirmative consent communication can lay the groundwork for future studies investigating why some may not perceive the same benefits as others and how individuals reconcile perceived rewards and costs of affirmative consent communication. Thus, a comprehensive understanding of both perceived rewards and costs is foundational to investigating ambivalent attitudes towards consent communication and could also play an important role in promoting affirmative consent communication. Accordingly, the first goal of the current research was to investigate both perceived rewards and costs of affirmative consent communication.

Our second goal relates to the context in which affirmative consent communication occurs. Most past work on consent communication, including perceived costs to consent communication, has been done in university/college samples (see review by Muehlenhard et al., 2016). This focus is warranted given such factors as the high rates of sexual assaults on college and university campuses (Fedina et al., 2016). However, less is known about affirmative consent in adult sexual relationships outside of institutions of higher education (despite the prevalence of sexual assault in community settings; Muehlenhard et al., 2017), so we wanted to extend this past work by investigating perceived costs and rewards to consent communication in community samples.

Our focus on perceived costs and rewards of affirmative consent communication is influenced by two theoretical perspectives. The first theoretical influence on the current study is a model of sexual health behaviours developed by Fisher and Fisher (1992), the Information-Motivation-Behavioural Skills (IMB) model. This model has been used to investigate a range of sexual health behaviours, such as HIV risk behaviour (Fisher et al., 2006) and prevention of adolescent pregnancy and STD infections (Fisher, 1990). More recently, it has been applied to investigate affirmative consent behaviours (Shumlich & Fisher, 2019). Below, we review the basic tenets of the IMB model and discuss how our investigation into perceived rewards and barriers to affirmative consent communication is situated within the IMB model. In addition, our work on perceived costs of consent communication is influenced by theoretical writing of Metts and Cupach (1989) where the authors detailed potential factors that can serve as barriers to sexual communication.

## Understanding consent through the Information-Motivation-Behavioural Skills lens

Shumlich and Fisher (2019) applied the Information-Motivation-Behavioural Skills Model (IMB model) to investigate the disconnect between legal or administrative standards for consent and normative consent practices. Although we do not apply the same legal or administrative lens in the current study, this model informed our understanding of the types of barriers and rewards that were likely to emerge. According to the application of the IMB model (Shumlich & Fisher, 2019) to sexual consent, three broad factors influence the expression of sexual consent: sexual consent information, sexual consent motivation and sexual consent behavioural skills. Sexual consent information includes awareness of laws and regulations relating to consent, knowledge of behaviours that may be required to unambiguously express consent, as well as knowledge of the means to decode behaviours signalling consent. Past research on this factor has shown that individuals vary in terms of their knowledge of what constitutes affirmative consent and can report contradictory information of what comprises consent (Beres, 2007). *Motivation* to engage in consent communication includes both personal and social motivations; the decision to engage in affirmative consent behaviours depends on the balance of these perceived rewards and costs (Shumlich & Fisher, 2019). *Personal motivations* include individual beliefs about the perceived impact/outcomes of the behaviour and the subjective evaluation of those outcomes. *Social motivations* refer to an individual’s perceptions of social outcomes for enacting or failing to enact consent communication, such as ostracism from one’s social circle. According to the IMB model, whether *information* and *motivations* will lead to affirmative consent behaviours will depend on whether the individual possesses the requisite *behavioural skills* to engage in consent communication (Shumlich & Fisher, 2019).

## Barriers to sexual communication

To better understand the types of barriers individuals can experience when engaging in affirmative consent communication, we also turn to the theoretical work by Metts and Cupach (1989). Although their work focused on general sexual communication and was not focused specifically on consent communication, it has the potential to inform our understanding of perceived barriers to consent communication. They noted that sexual communication may be avoided in an effort to avoid experiencing negative feelings such as embarrassment, shame, anxiety and guilt. Further, they theorized that another important source of avoidance may be fear of impact of sexual disclosure on the relationship; for instance, sexual communication could reveal incompatibilities between partners or evoke feelings of jealousy or anger in the partner. Based on this theoretical work as well as other empirical studies on barriers to sexual communication, Rehman and colleagues (2019) developed and validated a measure where they identified three categories of threat that could influence an individual’s willingness to engage in sexual self-disclosure with their partner: threats to *self*, threats to *partner* and threats to the *relationship*. The authors found that each of these types of threats was associated with lower relationship and sexual satisfaction.

## Sexual consent barriers and rewards in the extant literature

Shumlich and Fisher (2020) examined affirmative consent communication through the lens of the IMB model, using a mixed-method approach that included quantitative measures and focus groups. Interestingly, they found that the majority of individuals in their sample conceptualized consent in a manner that was consistent with the standards of affirmative consent communication (e.g. consent should be ongoing, reciprocal, unambiguous and informed) but that only few participants in their sample had explicit awareness of the term *affirmative consent*. This study also stands out because the authors investigated both positive and negative motivations to engage in affirmative consent. Their findings revealed that individuals’ willingness to engage in affirmative consent communication was strongly influenced by what they perceived to be the outcomes of the consent communication. In contrast to indirect communication, participants viewed direct consent practices to be disruptive to the flow and experience of sexual activity, expressed the concern that affirmative consent communication would lead their partner to view them as only interested in sex and/or inexperienced and worried that affirmative consent would increase the probability of being rejected. Participants also felt that there is more opportunity for face-saving in the context of indirect communication. Importantly, participants described numerous positive outcomes of affirmative consent communication and noted that direct and clear communication about consent reduces uncertainty, saves time and demonstrates respect for the partner. They further reported that affirmative consent communication can facilitate other types of sexual communication, such as discussion of sexual preferences. In the current study, we build upon this work by using a different qualitative approach, open-ended questions, to assess perceived costs and rewards of affirmative consent communication. In their study, Shumlich and Fisher (2020) used focus groups to assess participant responses and while such a methodology can provide rich data, it can also negatively impact an individual’s willingness to share their opinions, particularly on sensitive topics and if the opinions do not conform with what others have shared or what is in contravention of social norms (Smithson, 2000).

A recent study by Piemonte and colleagues (2020) investigated the association between verbal sexual consent and sexual quality. The researchers presented participants with written erotic stories, manipulating the presence versus absence of affirmative verbal consent in each story. Participants provided their evaluations of each story’s appeal and the extent to which they viewed the story as sexy. The presence or absence of verbal consent did not significantly affect overall judgements of the stories, suggesting that verbal consent is not detrimental to the quality of a sexual interaction. However, the researchers noted that characters in the narratives seamlessly integrated verbal consent into their interactions, whereas this process may be more difficult or disjointed in real-world sexual interactions. Presently, no studies have explored potential nonsexual benefits to consent communication, such as personal empowerment or relational improvements. Although there is a paucity of research on the rewards of sexual consent communication for individuals and couples, other forms of sexual communication have been shown to confer several advantages. For instance, openly discussing sexual problems with one’s partner and constructive responses from the partner are correlated with increased sexual and relationship satisfaction (Fallis et al., 2013). It is plausible that sexual consent communication may provide similar relational benefits to other forms of sexual communication. In addition to its potential role in preventing sexual assault, consent may also be associated with unique benefits such as feelings of safety with a particular partner. The role of more direct, process-oriented consent practices in promoting sexual pleasure has also been studied in specific sexual contexts such as within kink and BDSM communities (e.g. Wignall, 2020).

## The current study

The goal of our study was to elucidate the specific factors or outcomes that lead people to perceive sexual consent as challenging and rewarding in their personal relationships. Informed by the IMB model (Shumlich & Fisher, 2019) and theoretical work by Metts and Cupach (1989), we expected participants to describe consent as having effects on their emotions, relationships and sexual experiences, in addition to difficulties enacting specific behavioural skills. We implemented an open-ended, qualitative approach in order to achieve this goal and to obtain a broad spectrum of responses.

## Method

### Participants and design

Participants (*N* = 231) were recruited for an online survey through Amazon’s TurkPrime service. We required that participants be at least 18 years of age, located in the United States and in a current sexual relationship of 1 year or less in duration. These data were collected as part of a larger study exploring consent experiences early in relationships; this focus was based on previous literature outlining the important role sexual consent is presumed to play early in relationships (Humphreys, 2007). In order to obtain a more diverse sample with regard to sexual orientation and relationship type (i.e. same-sex and mixed-sex), we uploaded two tasks to TurkPrime: One which was shown only to individuals who identified as heterosexual in their TurkPrime profile and one which was shown to individuals who identified as gay, lesbian, bisexual or other. The recruitment and survey materials were identical for both groups.

Initially, 266 responses were obtained, but 26 participants were removed due to careless responding and failing validity checks, and nine participants did not meet the eligibility criteria. The survey consisted of open-ended questions as well as multiple-choice and Likert-type items that are not relevant to the current study.

Participants ranged in age from 18 to 68 with a mean age of 34.32 years and a median age of 33.00 years (*SD*_age_ = 9.69 years). Most of the sample was White (*n* = 178; 77.1%). Participants also identified as Black or African American (*n* = 20; 8.3%), Hispanic or Latino (*n* = 16; 6.7%), East Asian (*n* = 9; 2.8%), South Asian (*n* = 2, 0.8%) and Other Asian (*n* = 2, 0.8%). Four participants indicated they belonged to a race not listed in the questionnaire.

Participants reported both their sex assigned at birth and gender identity. For 98% (*n* = 227) of participants, sex assigned at birth and gender identity were consistent (i.e. participants were cisgender). Two participants were women assigned male at birth (i.e. trans women). Two other participants identified as a nonbinary female and a genderfluid female. All analyses were conducted using participants’ gender identities, regardless of whether they were cisgender, transgender or nonbinary (139 women, 90 men and two nonbinary/genderfluid).

Our sample was predominantly heterosexual (*n* = 167; 72.3%). Approximately one-fifth of the sample was bisexual (*n* = 47; 20.3%), 3% were gay or lesbian (*n* = 7), 2.2% were pansexual (*n* = 5) and 1.7% were asexual (*n* = 4). Most participants (*n* = 206; 89.2%) reported that their relationship was mixed-sex and the average relationship length was 8.31 months (*SD* = 2.41 months). Two-thirds of participants reported they were in a committed and exclusive relationship (*n =* 156; 67.5%). The remainder considered themselves to be in either a casual and exclusive (*n* = 37; 16.0%), casual and nonexclusive (*n* = 22; 9.5%) or committed and nonexclusive relationship (*n* = 16; 6.9%).

Participants also reported their education, income and employment status. Participants reported an average of 15.35 years of education starting with Grade 1, with a range of 7–24 years (*SD* = 2.16 years). Most participants (*n* = 193; 83.5%) were employed full time, with the remainder being part-time employees (*n* = 28; 12.1%), currently unemployed (*n* = 8; 3.5%), temporary/seasonal workers (*n* = 1; 0.4%), or retirees (*n* = 1; 0.4%). Participants’ annual household income was reported as follows: 20 participants (8.7%) reported an income between $5000 and $19 999 per year; 47 participants (20.3%) reported an income between $20 000 and $39 999 per year; 69 participants (29.9%) reported an income between $40 000 and 59 999 per year; 52 participants (22.5%) reported an income between $60 000 and $79 999 per year; 21 participants (9.1%) reported an income between $80 000 and $99 999 and 22 participants (9.5%) reported an income above $100 000.

### Materials

Participants responded to two open-ended items about their perceptions of sexual consent difficulties and advantages. Prior to answering these questions, participants were provided the following definition of affirmative consent: ‘*Sexual consent is defined as the ways that partners freely agree to sexual activity and actively communicate their willingness to participate in sex, including words, actions, gestures, facial expressions, etc*.’ This definition was based on a synthesis of definitions of consent in the literature (see Beres, 2007) and was reviewed by the research team to ensure quality. We aimed to provide a comprehensive definition of consent that would emphasize the process-oriented and communicative nature of sexual consent (as opposed to internal feelings) without restricting the types of communication that were considered appropriate (e.g. explicit verbal communication only) so that the definition would adequately capture the experiences of participants.

In addition, the following instructions were written prior to the open-ended questions: ‘*The following questions ask you to reflect on your experience communicating consent in your current and past sexual relationships. Like any form of communication, people can have both positive and negative experiences with providing and obtaining sexual consent. There are no right or wrong answers*’. They were then asked to identify ‘*the most negative and/or difficult aspect of sexual consent communication*’ and ‘*the most positive and/or rewarding aspect of consent communication*’.

The validity checks employed included three attention check items embedded throughout the survey (e.g. ‘Please select 4 if you are paying attention’), a Captcha screener for potential automated responses and a post-survey questionnaire that asked participants if they responded carefully and whether we should use their responses (while clearly stating that their remuneration would not be penalized or withheld). Participants who failed any of these checks were removed.

### Coding of qualitative responses

An inductive content analysis approach was used to analyse the data (Hsieh & Shannon, 2005). In this bottom-up approach, category development is tied to and guided by data and the coding scheme is developed through an iterative process. A senior research assistant and the lead author separately reviewed all responses and generated lists of barriers and rewards. Together, the lead author, senior research assistant and principal investigator reviewed the lists to identify areas of overlap/redundancy or disagreement. These lists were combined into a coding manual containing definitions and examples for each barrier and reward (see Supplementary Information). For each open-ended question, approximately half the sample mentioned multiple barriers or rewards; therefore, each response could receive more than one code, depending on the number of distinct barriers/rewards mentioned by the participant. For example, if a participant referred to both consent ruining the mood of a sexual interaction and making them feel guilty or ashamed, their response would be coded under two barriers: *reduces sexual quality* and *negative emotional reactions (self)*, both of which are described in more detail below*.*

Research assistants were trained on this coding scheme and coded the first 50 responses as a training exercise. A subsequent training session was held to address any concerns or conflicts; coder feedback indicated that no changes to the coding manual were required. Then, research assistants were asked to code the remaining responses; each response was coded by at least two coders. If the coders disagreed, the senior research assistant and lead author discussed the response to determine the final codes. Given that all discrepancies were reconciled through discussion, we did not calculate interrater reliability (McDonald et al., 2019).

## Results

The barriers and rewards that were identified by participants reflected four general content areas: (1) Communication Quality, (2) Relational and Emotional Experiences, (3) Sexual Quality and (4) Safety and Coercion; these are described in more detail below. Across each of these broader content areas, participants identified 15 unique barriers and 10 unique rewards (see Table 1). A few participants gave responses to the barriers item that did not fit within any of the content areas: eight participants reported that they believed that sexual consent is unnecessary and viewed it as an obstacle that prevented their engagement in sexual consent practices. Further, two participants provided barriers that could not be categorized due to lack of clarity. Notably, 4% of participants declined to explicitly report a reward associated with consent and 13% of the sample declined to report a barrier. We examined the specific reasons provided: For rewards, the most common reason was that the participant stated they did not have enough experience with consent to identify a reward. For barriers, two-thirds of those who declined to list a barrier reported that they had never had a negative experience with consent and others reported that they did not have enough experience with consent to identify a barrier.

**Table 1.** Perceived barriers and rewards associated with sexual consent communication.

Reported sexual consent barriers and rewards	*n*	%
(1) Communication Quality		
*Rewards*		
Clarification of expectations	97	42.0
Ease of communication	22	9.5
*Barriers*		
Potential for miscommunications	39	16.9
Refusing a sexual initiation from the partner	38	16.5
Awkward or unnatural	30	13.0
Difficult to initiate	23	10.0
Complicated by context	20	10.0
Receiving a refusal from the partner	13	5.6
Unresponsive partners	9	3.9
Lack of communication skill	7	3.0
Inexperience with consent communication	6	2.6
(2) Relational and Emotional Experiences		
*Rewards*		
Enhanced relationship quality	66	28.6
Relationship communication norms	14	6.1
Get to know partners better	12	5.2
*Barriers*		
Negative emotional reactions (self)	56	24.2
Negative emotional reactions (partner)	25	10.8
Reduced relationship quality	24	10.4
(3) Sexual Quality		
*Rewards*		
Enhanced sexual quality	45	19.5
Facilitates access to sex	18	7.8
Streamlined consent communication over time	3	1.3
*Barriers*		
Reduces sexual quality	29	12.6
Navigating sexual incompatibilities	28	12.1
(4) Safety and Coercion		
*Rewards*		
Sexual safety and respect for boundaries	71	30.7
Legal and/or social protection	5	2.2
*Barriers*		
Sexual boundary violations	51	22.2

### Communication quality and skill

Participants frequently discussed the quality, and particularly the clarity (or lack thereof), of consent communication in their responses. The most referenced consent reward was that consent communication allows the *clarification of expectations and avoidance of misunderstandings*. Participants wrote that through consent, partners develop a shared understanding of what sexual activities are permitted or not permitted in the relationship, which they explained could be more difficult to establish when active consent is not practiced: One participant wrote that it was helpful to have ‘boundaries set before a sexual encounter happens, so it’s not just heat of the moment’. Despite this, some participants found the *initiation of consent communication* challenging, including finding an appropriate time to discuss consent. For example, one participant wrote that the most significant challenge was ‘waiting for the right moment to actually ask’, while another indicated difficulty ‘knowing when it is time to address sexual consent communication and when it is too soon’.

Some participants described reduced confusion and stress in sexual interactions due to sexual consent communication, such as in the response ‘you have clarity on what the other person’s desires, limits, likes and dislikes are’. At the same time, some participants felt that having to explicitly ask about these topics was an indicator that something was wrong within the relationship. For example, one participant wrote: ‘If I was to the point of feeling like saying [something about consent], then I need to not be there. It isn't a natural thing to say’. These responses suggest that some participants believed that if direct consent communication needs to be used, it is a sign that the persons involved should not be having sex or that the partners lack maturity (i.e. verbal consent is a less sophisticated form of sexual communication).

Furthermore, many described the *potential for miscommunications* as a barrier to effective consent communication. We distinguished this from sexual boundary violations or coercion by the participant’s emphasis on unintentional errors or transgressions rather than deliberate or malicious acts. For instance, participants recounted instances where they found it difficult to interpret ‘conflicting signs’ or where a partner appeared outwardly to be consenting but was experiencing internal ambivalence (e.g. they ‘would make me feel they want to [have sex] one second but should have told me flat out that they were not ready’). Participants often highlighted that people come into sexual relationships with different expectations about what consent should look like and that this can cause miscommunication. For example, one participant wrote ‘people can make assumptions, just because something is a natural progression to you it might not be for someone else’ and another stated ‘I have sometimes misinterpreted someone’s shy nature for lack of interest, as I naively expected consent to be verbal’.

It appears that participants found that explicit and direct consent communication generally facilitated smoother and more accurate consent communication, as it creates opportunities to clarify ambiguous messages or prevent misunderstandings. Indeed, participants generally described miscommunications as occurring when they relied on nonverbal cues (e.g. facial expressions) or when there was a conflict between verbal expressions and body language (e.g. partner saying they want to continue while appearing hesitant; token resistance such as ‘playing hard to get’). At the same time, clearer forms of consent communication were described by many participants as cumbersome or immature, as noted above. One participant noted that directly asking about sex could come across as ‘creepy and pushy’, and another suggested that it could be ‘embarrassing’ to use specific language around sexual acts. For some individuals, it may be easy to interpret and clarify ambiguous messages (e.g. the 10% of participants who viewed the *ease of communicating* consent as a benefit in and of itself).

However, for those who are more indirect in their communication, direct questioning may be avoided due to a sense that it is *awkward or unnatural* (e.g. ‘It can be awkward when you have to keep asking whether you can do certain things’). A few participants also reflected on how their level of communication skill might affect the process of establishing consent. Some indicated their own *inexperience* with direct consent communication and either did not know what to say to obtain consent or fell into indirect forms of communication instead (e.g. ‘we haven’t really talked about it. We kinda just flirt until we decide we want to have sex’). Others attributed their inability to communicate effectively or assertively in particular situations to a *lack of skill*. For example, one participant wrote that they have ‘wanted to be firm and ended up accepting something [they] didn’t really want to do’ because they lacked knowledge of how to voice refusals. Participants who voiced lack of skill or experience as a barrier typically focused on the expression of consent (e.g. how to ‘express whether I’m consenting to what’s happening or not’, or ‘form all my thoughts into cohesive sentences’) rather than difficulty determining whether a partner is consenting.

About 17% of participants noted that they found *refusing a sexual initiation from their partner* difficult, whether in a new or an established relationship. Some participants noted that it was difficult to refuse a partner during sexual activity, such as if the participant had changed their mind about sex (e.g. ‘the most difficult is saying, after you already started to engage, that you’re not feeling quite right and would like to pause’) or wanted to set a boundary (e.g. ‘if you are already involved in a sexual activity and you don’t want to go further, it’s hard to interrupt the mood like that’). Participants also expressed concern about how their partners would react to refusals. For example, one participant stated: ‘I won’t be into it anymore, and it’s hard to communicate that change because I’m worried about hurting the guy’s feelings or having led him on’. Despite this fear, only a small minority (5.6%) of participants wrote that *receiving a refusal from their partner* would be difficult (e.g. feeling frustrated or rejected due to a refusal, finding it difficult to stop sexual activity if the refusal comes during sex).

The relational and environmental *context* was considered by some participants to be barriers to implementing good consent communication. Participants expressed particular concern about sexual encounters when one or both partners are intoxicated. For example, one participant wrote, ‘In the past I have had guys who think that being drunk or buzzed changed the seriousness of the talk’ and another noted that ‘everyone’s tolerance levels can be different with drugs, and you may not know if a person is giving true consent’. Some participants wrote that consent was more difficult in their youth but became easier with age and experience. For example, one participant wrote, ‘I think when I was younger, I was perhaps embarrassed to say “no” and people took advantage of that’. Participants also noted that communicating consent was more challenging (and more frequently avoided) in early stages of a relationship.

Finally, participants noted that even when they themselves valued and felt comfortable with direct consent communication, it could be difficult to engage with *unresponsive partners.* That is, some participants found it difficult to establish consent ‘when the other person doesn’t open up and express themselves’, ‘freezes up’, or otherwise tries to avoid consent communication. Participants frequently described this as impacting the quality of sex or of the relationship, such as one who stated, ‘It’s much harder to have an intuitive relationship when your partner doesn’t want to communicate or is only interested in themselves’. Partner unresponsiveness was also attributed to partners being ‘too eager’ to have sex and feeling that discussions of consent are inconvenient as they interfere with the experience of sexual activity itself.

### Relational and emotional experiences

Participants described a variety of ways that consent communication had impacted their emotions and/or altered their relationships. Nearly a quarter of participants expressed concern that consent communication could cause personal *negative emotional reactions* (i.e. in the self). A variety of negative emotions emerged, including anxiety/worry, annoyance, fear, frustration, guilt and embarrassment. Such emotions were often attributed to the general prospect of consent communication and sexual communication more broadly, such as ‘feeling embarrassed about talking about different aspects of sex’ or anxiety related to sexual communication inexperience. Some participants described it as irritating to have a partner who ‘continually talks about sex’, whereas others found it frustrating to have a less communicative partner. Furthermore, participants described specific situations that caused negative emotional reactions; these included situations that compromised participants’ physical and emotional safety (e.g. experiencing coercion) but also more benign situations such as enquiring about introducing new sexual acts (e.g. anal sex), delivering negative sexual feedback (e.g. ‘after having unpleasant sex’, rejecting an advance or talking about lack of attraction/desire) or initiating sexual consent discussions for the first time in a new relationship.

Some participants also described concern that consent would cause *negative emotional reactions for partners*. This barrier was less common (cited by 10.8% of the sample) than worries about own emotions, but a range of possible negative partner emotions were described including anger, shame, annoyance or offence. Specifically, participants described actual or potential situations in which partners became offended, uncomfortable or irritated that the participant wanted to directly communicate consent (e.g. because it contradicts the social norm of more passive forms of consent). For example, one participant described past instances of consent communication as feeling ‘like a teacher talking to a student, or a mother to a child’ and worried that she ‘annoyed’ her partners with such communication. Another wrote that she was concerned that her partners might feel ashamed or nervous if the subject of consent is brought up. Partners’ perceived and/or expressed discomfort with openly communicating consent was frequently cited as a reason to either avoid sexual consent communication or end a sexual relationship altogether. Additionally, several participants described their partners becoming angry in the face of sexual refusals or sexual boundary-setting (see Safety and Coercion below). Within the rewards item, other participants described positive emotions that occurred when both partners communicated consent directly; these included feeling ‘accepted’, ‘comfortable’, ‘at peace’, and ‘respected’. Most of these emotions were relational in nature (e.g. feeling more respect or trust with a current partner), so we categorized them as *enhancing relationship quality.*

Participants recounted other ways that direct consent communication seemed to *enhance relationship quality* as well*,* such as through promoting mutual respect and reciprocity: Participants expressed sentiments such as ‘I like it when people ask me explicitly, because it makes me feel like they care about my experience, not just their own’. Building further on themes of mutuality and trust, a few participants felt that consent communication *established communication norms* for future interactions in their relationship and discussions of other topics that may be difficult to bring up (e.g. ‘feeling reassured that consent is appropriate to discuss, creating open channels to discuss other subjects’). Further, participants felt that explicit consent communication helped them to *get to know partners better*, including understanding each other’s values or learning about sexual preferences, particularly early in the relationship. For example, one participant wrote that consent helps partners ‘understand one another at a different level and that [they both are] there for each other’. However, in contrast, those who thought consent *reduces relationship quality* often viewed the need for directness in consent as ‘making it seem like you do not trust the other person’. Participants also expressed concern that directly refusing sexual activity could not only cause conflict (e.g. an argument), but could also lead to the end of a relationship (e.g. ‘I’m afraid if I vocalize that I want to wait, then they’ll react negatively and not want to be with me anymore’).

### Sexual quality

In addition to relational quality, participants indicated that the ways in which consent is practiced (e.g. the type of signals and the level of skill involved in providing, obtaining and interpreting consent) could affect the quality of their sexual interactions. Again, there was variability within the responses regarding whether direct consent *enhanced sexual quality* (e.g. ‘without the communication aspect, there is something less “sexy” about it’) or *reduced sexual quality* (e.g. ‘asking whether you can do things can take away from the spontaneity of sex’). Several participants were hesitant to practice consent because it might ‘ruin the mood/moment’. At the same time, many participants felt that open consent communication made them feel more at ease or ‘relaxed’ during sex. A few participants included negotiation of safer sex practices (e.g. condom use) as a component of a consent that could ‘kill the mood/vibe’ as well.

Sexual compatibility was another common theme within responses. A perceived limitation was that consent could *reveal incompatibilities* between partners that could threaten or jeopardize the sexual relationship. Direct consent was considered to make incompatibilities explicit, whereas with indirect communication, they could be avoided, ignored or bypassed. The types of incompatibility referenced included disagreement about what constitutes consent, such as one participant’s response that consent can be challenging ‘when the other person doesn’t feel or believe that consent can/should be able to be revoked at any point’. Other sources of conflict included difficulty agreeing on sexual boundaries and safer sex practices (e.g. ‘the only negative I can think of is the subject of condoms and or birth control’.).

Yet, consent also provided opportunities for partners to learn that they are compatible and share similar desires for their sexual relationship. When this was the case, consent was considered to have a variety of beneficial effects on the sexual relationship. In addition to finding consent communication itself sexually arousing (e.g. ‘some of the responses I received over the years were downright hot’), participants noted that consent allowed for sexual experimentation and improved sexual communication more generally. For instance, one participant wrote: ‘Having open discussions about consent means that we can safely experiment with new things which is an absolutely fantastic part of our sex life’. Finally, two participants noted that consent becomes *streamlined over time,* shifting from more explicit and direct communication towards more nonverbal and implicit approaches that signify the ability to pick up on their partner’s preferences and subtle communicative signals. Altogether, these rewards emphasized a broader view of consent communication that includes not only communication during sexual activity, but between sexual encounters as a way to enrich their sexual relationships and perceived intimacy.

On a more practical level, a few participants considered consent to be rewarding because it *facilitated access to sex* (e.g. ‘being able to know, at the beginning, that sex is going to happen’ or ‘when we finally get into the action, and all goes as it should’). Participants who cited this as a reward conceptualized consent as either a single event that takes place at the beginning of sexual activity or a lack of refusal (e.g. ‘she never says no at all’), in contrast to the more process-oriented nature of affirmative consent.

### Safety and Coercion

Themes of *sexual safety and respect for boundaries*, as well as past and potential future instances of *sexual boundary violations*, were evident within the responses. Regarding safety, almost one-third of participants wrote that consent conferred such benefits as ‘knowing we won’t pressure each other’, or ‘not having to worry that there is any coercion going on’*.* We distinguished this from the reward of ‘clarifying expectations’ by the emphasis on protection against deliberate, rather than unintentional, transgressions (i.e. where one partner behaves in a malicious or coercive manner). Some participants also felt more comfortable stopping sexual activity or voicing concerns in relationships where clear consent communication was an established norm. For example, ‘being able to discuss consent during an encounter with a partner you can trust to listen means that if something suddenly becomes “not okay” it can be conveyed’. A few participants also noted that consent could protect them from social or legal consequences (i.e. in the face of ‘false accusations’), including statements such as ‘it gives you the relief of knowing that this person probably won’t turn around and say that you took advantage of them’, and more specifically, ‘no fear of lawsuit/charges’. These participants prioritized the legal function of consent and did not emphasize or mention the role of consent in ensuring physical and psychological safety for all involved.

However, as much as consent was viewed as vital to feelings of safety and preventing violations, a relatively large number of participants (*n* = 51; 22.2%) unfortunately described instances where their consent or lack thereof had not been respected and, in some cases, when they had been unable or unwilling to understand their partner’s sexual refusals. Frequently, participants described instances where they had clearly communicated nonconsent to sex, but their partners persisted until they gave in (e.g. ‘I explicitly told a few partners no and they persisted anyway. I have also been guilted into sex a number of times’). Participants also described experiences where consent was assumed despite not being clearly communicated, such as due to sexual history (e.g. ‘I didn’t necessarily want to have sex, but it was implied that I did because we have had sex in the past’) or because consent to a specific sexual act (e.g. kissing) was interpreted as ‘blanket’ consent to other acts.

Although most descriptions of boundary violations occurred with past partners rather than within participants’ current relationships, a history of being sexually coerced was often highly salient and influential in future sexual interactions, such as a participant who described an instance of having her boundaries violated and wrote, ‘that fear still kind of lingers with me no matter what’. Further, participants often described finding it difficult to voice refusals due to fears ranging from ‘not knowing if the other person is going to respect what I’m saying’ to ‘wondering if a partner may react violently’.

In addition to participants having their own boundaries violated, a few responses indicated irritation with having to respect another person’s boundaries or refusals. For example, a few participants wrote that it was frustrating to have a partner ask to stop ‘in the middle of having sex’ when the participant themselves wished to continue. For the most part, these participants indicated that they respected their partners’ refusals in the moment (e.g. ‘you have to do as they wish’) and considered the barrier to be about managing their own emotions or increasing their awareness of nonconsent cues (e.g. ‘I felt guilty and realized that I should have picked up on signals that I did not’). At the same time, several participants indicated that partners had become angry and physically or verbally coercive when refusals were voiced, suggesting that the behavioural response towards refusals remains an important consideration.

#### Exploratory analyses

Though we did not have specific predictions about gender, we conducted exploratory chi-square tests to see whether there were gender differences in the endorsement of barriers and rewards, given that men and women typically have different assigned roles and experiences with regard to consent (Jozkowski, Peterson, et al., 2014a). After correcting for multiple comparisons using the Bonferroni method, two barriers (sexual boundary violations and refusing a sexual initiation from the partner) were more frequently endorsed by women than men (χ^2^(1, 229) = 12.90, *p* < .001 and χ^2^(1, 229) = 18.84, *p* < .001, respectively). There were no differences for sexual consent rewards (all *p*s >.05)

## Discussion

Informed by the IMB Model (Shumlich & Fisher, 2019) and prior work on emotional threats in sexual communication (Metts & Cupach, 1989; Rehman et al., 2019), we collected data from community participants to explore lay perceptions of affirmative consent barriers and rewards. Prior work had examined individual-level barriers to affirmative consent communication (e.g. Curtis & Burnett, 2017; Shumlich & Fisher, 2020), but there has been limited research on the perceived advantages of such communication relative to more passive, indirect communication about consent (with Marcantonio et al., 2020, Piemonte et al., 2020 and Shumlich & Fisher, 2020 as notable exceptions). Further, most investigations have focused on postsecondary student populations that are predominately heterosexual (Muehlenhard et al., 2016); we aimed to understand whether a community sample would report similar challenges to what has already been reported in the literature.

An analysis of the themes in our qualitative data shows that all of the barriers to open sexual communication identified by Metts and Cupach (1989) were also cited by our study participants as potential costs of affirmative consent communication; these include the worry that consent communication may *reveal incompatibilities* that will threaten the relationship, that it can be *difficult to articulate*, that discussing consent communication is *unnecessary*, and can evoke *negative feelings in oneself and one’s partner*. Shumlich and Fisher’s (2019) application of the IMB model, a model that has been used to study a range of sexual and reproductive health behaviours (e.g. Camilleri et al., 2015), to sexual consent communication further develops and formalizes these perceived barriers in a number of ways. First, they outline the role of both perceived rewards and costs of consent communication. Further, they postulate that the balance of rewards and costs is the key motivational component underlying sexual consent communication. Consistent with the IMB Model (Shumlich & Fisher, 2019), our findings indicate that the decision to engage in affirmative consent behaviour likely depends not only on the behavioural skills that individuals possess, but also their beliefs about whether sexual consent will compromise or enhance their relationship, whether it will cause negative experiences for the self or partner (e.g. negative emotions, sexual rejection) and whether it contravenes acceptable social and/or relationship norms. Experiences with sexual violence and coercion were also described as influential in future decisions about consent and general comfort with affirmative consent. Participants reflected on past experiences that made them hesitant about affirmative consent or, alternatively, that established affirmative consent as a strongly held value, suggesting that sexual experience and partner feedback play a key role with regard to consent decision-making that can sometimes conflict with educational or public health messages.

Our qualitative analyses suggested that participants’ perceived costs and rewards of consent communication can be conceptualized through four broader categories. Below we describe the range of themes that related to each category, noting the considerable variability in participants’ conceptualizations of perceived rewards and costs associated with consent **communication, and** speculate on possible reasons for this variability. In the subsequent sections, we discuss the implications of the observed themes for education, research and further theory development on affirmative consent communication.

### Communication quality

Consistent with previous literature identifying consent as ‘awkward’ (e.g. Curtis & Burnett, 2017; Shumlich & Fisher, 2020), many participants in our sample viewed the expression of affirmative, direct and/or verbal consent as clumsy, unnatural or aberrant. However, as much as affirmative consent posed challenges in many participants' eyes, others highlighted that indirect consent communication is rife with the opportunity for miscommunications (making affirmative consent preferable). Participants’ divergent perceptions of affirmative consent appear to reflect the lack of a universally accepted standard for sexual consent practices, which likely also fuels the miscommunications described by participants. Though participants in our study and others value affirmative consent in theory (e.g. Curtis & Burnett, 2017), in practice it may not be considered widely socially acceptable; participants frequently noted concern about how their partners might respond to initiations of sexual consent discussions, particularly early in the relationship when the partner’s attitudes may be unclear. The perceived risks of a negative partner reaction (e.g. relationship conflict, sexual rejection) may contribute to avoidance of explicit communication, potentially even outweighing other risks such as sexual safety for a subset of individuals. Avoidance of consent communication in the early stages of a relationship is important to address given that this is the context in which partners would be the least familiar with each other’s consent signals.

Additionally, we observed that many participants referenced difficulty with consent due to lack of skill or experience, including those who mentioned that they found consent difficult when they were young. Implicit within these statements is the notion that consent communication skill is developed through successive sexual experiences rather than through comprehensive sex education or modelling in non-sexual contexts. An analysis of sexual curricula indicates that in the United States, consent is unlikely to be taught in kindergarten through grade 12 (Willis et al., 2019), meaning that young people may go into their first sexual experiences without a clear understanding of consent and how it ought to be practiced. Consent is most often learned through channels such as the media, internet and peer groups, which may result in inadequate or distorted perceptions of consent and limited skill for sexual consent communication (MacDougall et al., 2020).

We observed that participants described more difficulty expressing their own consent or asking about consent, rather than difficulty interpreting consent signals or respecting a partner’s decision. Difficulty with expressing consent versus interpreting it may be reflective of the lack of affirmative consent modelling in popular media and educational programmes. However, many of our participants described experiences of sexual coercion and of their refusals being ignored by partners. Additionally, prior work on sexual refusal indicates that people possess the ability to interpret subtle verbal and non-verbal sexual refusals (O’Byrne et al., 2006). This suggests that although some participants described difficulty expressing nonconsent or sexual refusal, a high degree of skill is not necessary to convey that one is not interested in sex. Other factors, such as attitudes towards sexual consent and acceptance of problematic cultural beliefs surrounding sexual assault (i.e. rape myths), also need to be considered in the context of sexual coercion (Trottier et al., 2021).

### Relational and emotional experiences

Participants positioned sexual consent as a foundational relational experience, particularly in new relationships, suggesting that compatibility regarding how consent should be practiced has potentially long-term implications for sexual relationships. This is consistent with sexual script theory, which suggests that early sexual interactions create a template for future interactions within that relationship (Gagnon, 1990). Moreover, early positive sexual consent communication was considered to create an atmosphere of trust, respect and safety (both sexual and emotional). For some participants, the effects of early sexual consent experiences within a relationship also provided information about nonsexual aspects of the relationship: Consent was occasionally considered an opportunity to get to know more about a partner’s values and communication style, and it ‘set the stage’ to talk about other serious topics. Previous studies have similarly found that open sexual communication contributes not only to sexual satisfaction, but also to overall relationship satisfaction (Montesi et al., 2010). Consent may play a unique role in communicating information about values such as mutual respect, reciprocity and prioritizing sexual safety over sexual entitlement or pleasure.

Despite these promising advantages of consent, not all participants agreed that affirmative consent was beneficial for relationships or for the self. The most common challenge, expressed by nearly a quarter of participants, was concern that consent communication could evoke negative emotions in the self, including feelings of anxiety, shame and embarrassment. This is consistent with the finding by Rehman and colleagues (2019) that threats to the self were activated more strongly during sexual conflict communication (compared to threats to the partner or relationship, although all three types were activated to some degree). However, threats to the partner and relationship were also described (e.g. that consent reduces trust or may hurt partners’ feelings), indicating that several different types of threat exist for consent communication and may need to be uniquely studied and/or targeted. The inability to constructively manage negative emotions appears particularly important in the context of sexual refusal, as past work has demonstrated that emotions such as anger, confusion and rejection can increase the likelihood of verbal coercion following such a refusal (Wright et al., 2010).

### Safety and coercion

Many participants described violations of consent or non-consent as a challenge (e.g. experiences of coercion or partner ignoring refusals). This barrier was among the most frequently cited in the responses. The reasons a partner may violate a sexual boundary can vary from the relatively benign (e.g. partner is working from a traditional sexual script and believes they can proceed until vocal, outright refusal) to the more egregious (e.g. wilful and knowing violation). This category of responses speaks to how one partner’s ignoring of refusal cues can make it difficult or challenging for the other person to initiate such communication in the future. As a result of these experiences, some participants described a lingering fear or apprehension that they carried into new relationships; work by other scholars echoes that sexual trauma can alter sexual consent practices even in future healthy relationships (Mark & Vowels, 2020). Additionally, sexual compliance, or acquiescing to unwanted sexual activity, is more likely when one’s partner has exerted pressure for sexual activity in the past (Vannier & O’Sullivan, 2010).

### Sexual quality

Affirmative consent was considered by different sets of participants to either ‘ruin’ or bolster sexual experience. Previous studies have suggested that affirmative consent is believed to interrupt the flow of sexual experience or decrease sexual arousal and enjoyment (e.g. Curtis & Burnett, 2017; Shumlich & Fisher, 2020), a belief that was endorsed by some of our sample. However, a greater number of our study participants reported the perspective affirmative consent can *enhance* sexual enjoyment, in addition to facilitating other types of sexual communication (as described by Shumlich & Fisher, 2020). In contrast to both of these perspectives, Piemonte et al. (2020) found that affirmative/verbal versus nonverbal consent had no impact on the perceived quality of sexual stories.

Lay beliefs that affirmative consent can be arousing or exciting contradict traditional sexual scripts and media representations of consent (Jozkowski et al., 2019), but are compatible with newer messaging around consent (i.e. ‘consent is sexy’). What drives the perception of explicit consent as erotic versus artificial? This is an open empirical question, but our results and the prior literature offer some insights. Piemonte et al. (2020) suggested that verbal consent may be viewed as ‘unsexy’ when participants lack skill and fluency in this type of communication. For those who experience negative emotions during sexual consent communication (as identified in our study), difficulty regulating these emotions may also interfere with the fluidity of consent communication and motivate avoidance.

Interestingly, participants expressed concern that affirmative, verbal consent would lead to revelations of sexual incompatibility (with regard to safer sex practices, sexual preferences, etc.). It is important to clarify that these incompatibilities would continue to exist and possibly compromise sexual quality regardless of consent practices; sexual consent simply makes disagreements explicit. One cost of openly discussing consent is that one may learn that their intuitions about their partner’s desires are misguided. This may, for example, threaten a person’s sense of sexual competence. In contrast, avoiding direct consent communication may serve to maintain one’s belief that they can correctly ascertain what their partner wants. However, when consent helps to reveal that partners are compatible, it may also provide opportunities to improve the sexual relationship (e.g. through experimentation), consistent with the literature on general sexual communication which suggests that such communication helps to maintain sexual satisfaction over time (Frederick et al., 2017).

Our findings corroborate those of Shumlich and Fisher (2020), who identified similar barriers to affirmative consent (e.g. effects on sexual quality, concerns about being rejected) and who highlighted some positive aspects of affirmative consent (e.g. increased clarity, feelings of respect). In addition to replicating their findings on the drawbacks of affirmative consent, we also shed light on the perceived pitfalls of *indirect* consent, which motivated clearer and more direct communication for a portion of our participants. Our work also directly contributes to a more expansive understanding of the ways that affirmative consent can be beneficial. Further, whereas Shumlich and Fisher (2020) studied a primarily heterosexual university student sample, the current study demonstrates that similar barriers are present in a community sample who represent a more diverse demographic in terms of sexual orientation.

### Gender differences

We conducted exploratory analyses to identify whether there were any gender differences in the endorsement of various barriers and rewards. More women than men endorsed boundary violations as a barrier to consent communication. This is consistent with the majority of research which concludes that women are at an increased risk for sexual assault and coercion (Conroy & Cotter, 2017). Women were also significantly more likely to report difficulties refusing their partner’s sexual initiations. In addition to women’s heightened concerns about safety (i.e. worrying that their partner will not respect a sexual refusal), it may be that men did not fear refusing a partner because within the traditional sexual script, they are typically the ones expected to initiate sexual activity (Jozkowski & Peterson, 2013), so they rarely have occasion to refuse their partners but more often put themselves in a position to be refused.

### Theoretical implications

As applied to sexual consent, the IMB model (Shumlich & Fisher, 2019) posits that *sexual consent information* and the *motivation* to engage in consent communication indirectly influence sexual consent communication through the mechanism of *behavioural skills*. In the current study, all three aspects of the IMB model were identified as barriers to affirmative consent communication, but the motivational aspects were particularly prominent (e.g. effects of consent on the relationship, on own and partner emotions, on sexual satisfaction/enjoyment and on perceptions of safety). It may be that the methodology used in the current study was more conducive to participants remembering emotional barriers they have experienced. Research on emotions and memory has demonstrated that emotional memories, particularly those relating to negative emotional experiences, are likely to be retained in greater detail than memories of neutral or positive experiences (see review by Kensinger, 2009). However, if the current findings are replicated with different methodologies, this would lend credence to the idea that emotional barriers are more frequently experienced, as compared to information or behavioural skills deficits, when it comes to practicing affirmative consent.

Compared to some other types of sexual health behaviours, consent is a highly dyadic, interdependent process. That is, effective consent communication relies on both partners’ engagement in the mutual process of communicating their intent and interpreting the other person’s consent cues. Integrating the IMB model with the Actor-Partner Independence Model (APIM; Cook & Kenny, 2005) would allow us to test how both partners’ consent knowledge, motivations and behavioural skills interact with each other to influence sexual consent behaviours. For example, it may be that partners can compensate for gaps in one another’s knowledge of consent or that one partner’s mood or personality features influences the other’s motivations (e.g. being pulled to communicate less directly upon perceiving one’s partner is anxious). Such a model would be more closely reflective of how consent communication occurs in everyday interactions.

### Implications for education and prevention

Though further investigation is needed, these findings have implications for initiatives that aim to increase affirmative consent communication and for sexual assault prevention initiatives more generally. First, sexual assault prevention initiatives typically target such outcomes as behavioural skills, rape myth acceptance, belief in gender stereotypes and intentions to seek consent (Hovick & Silver, 2019; Paul & Gray, 2011; Thomas et al., 2016). These outcomes are undoubtedly valuable in promoting affirmative consent practices. However, the current study suggests that the emotional challenges associated with consent communication also need to be addressed, particularly the management of negative emotions such as shame or guilt, and of fears that relationship quality will suffer. Moreover, the multitude of barriers that were identified suggests that consent interventions cannot be one-size-fits-all: People vary in the types of barriers that they experience, and programming should reflect this broad range of concerns.

Sexual consent interventions must also take the *context* of consent communication into account, with particular attention paid to the conditions of intoxication and/or new relationships. Many sexual health programs address the link between alcohol and impaired sexual decision-making, particularly in postsecondary educational settings that have established norms around alcohol and ‘party culture’ (Muehlenhard et al., 2016). The evidence from the current study reinforces this focus and demonstrates that within the context of sexual consent communication, alcohol use is considered an impediment. Given that early relationships were cited as another particularly challenging context, some interventions could focus on this stage, including practical skills to negotiate consent and tolerate the anxieties associated with refusing a partner. Whereas concern about refusing a partner was among the most endorsed barriers, concern about being refused by one’s partner was mentioned by a much smaller number of participants. This suggests that the perceived cost of refusing a partner might be higher than the actual cost (i.e. most people will be accepting if their partner is not in the mood to have sex at a particular time). Educators might capitalize on this by *normalizing* sexual refusal, such as by reiterating that it is both common and permissible to refuse a sexual initiation, even in an established relationship.

Discussion of sexual consent rewards would likely also substantiate sexual consent interventions, perhaps making them more persuasive to their audiences. Though many participants viewed consent as detrimental to sexual quality and aspects of the relationship such as trust, a greater number of participants felt the opposite: that consent facilitated a more enjoyable sexual experience and increased the level of relational intimacy between themselves and their partners. The relational aspects of consent, such as its potential to foster trust and respect, can be emphasized alongside benefits to sexual quality.

### Strengths and limitations

The current study is the first to describe lay perceptions of rewards or incentives to sexual consent. This is an important direction as it merges a pressing social issue (i.e. the adoption of affirmative consent practices to prevent sexual violence) with newer, promotion-focused discourses of sexuality (e.g. Beres, 2007). Over time, study in this area may lead to consent being viewed as a means to greater sexual well-being in addition to its crucial function of ensuring psychological and physical safety for all parties in a sexual interaction.

Most research on barriers to sexual consent has been conducted with young people (primarily in college/university settings). In the current study, participants were recruited via MTurk and represented a broader demographic. Therefore, the findings establish that barriers identified in the postsecondary setting (e.g. awkwardness, complications due to alcohol; Curtis & Burnett, 2017; Muehlenhard et al., 2016) are also relevant in other demographic groups. Though most interventions occur in postsecondary settings, nonstudents are equally at risk for sexual assault (Muehlenhard et al., 2017) and therefore consent education needs to be available more widely and to a broader target audience.

Because we asked participants to write about the *most* challenging and *most* rewarding aspect of sexual consent communication, we were unable to assess the coexistence of multiple barriers/rewards, the relationship between perceived barriers and perceived rewards or the relative salience of each of these barriers/rewards. For example, a participant who wrote about negative emotions in the self might still experience a barrier such as reduced sexual quality, but not describe it in their response because it was less notable for them. Below, we discuss future research directions that are needed to address this limitation.

Our study is also limited in part due to some demographic data that were not collected. For example, we did not explicitly ask participants about whether they were current students or the type of educational credentials they held (e.g. earning a high school diploma, undergraduate degree or graduate degree). This is specifically relevant in the context of consent because, as outlined above, consent intervention primarily targets students, and it is reasonable to expect that students might differ from nonstudents in their understandings of and experiences with consent. Additionally, we did not collect information about whether participants had physical or intellectual disabilities, which could meaningfully impact their experiences with consent communication.

### Future directions

A productive next step would be to develop quantitative measures that allow us to test the *relative* strength of multiple barriers and the *relative* strength of multiple rewards. This would enhance our understanding of which barriers and which rewards contribute to variation in sexual consent practices and to identify individual differences that predict endorsement of certain barriers and rewards. For example, relative to other attachment styles, anxious attachment has been associated with increased willingness to consent to unwanted sex due to perceived threats to the relationship (Impett & Peplau, 2002).

Further investigation of the rewards or benefits of consent communication is also needed. Although participants perceived sexual consent as improving sexual and relational quality, it may instead be that sexual and relationship quality enhances consent communication. Therefore, where possible, future work should test the longitudinal, prospective association between sexual consent communication in relationships and relationship outcomes, such as sexual and relational satisfaction and feelings of trust/closeness.

## Conclusion

This qualitative investigation identified perceived barriers and rewards of sexual consent communication. Participant responses reflected variation in communication skill/quality, as well as discrepant perceptions of how consent could impact sexual and relational experiences. There was also variation in the extent to which participants viewed consent as protecting themselves and their partners; several participants recounted instances where their lack of consent was ignored despite direct and active communication on their part. These findings have important implications for sexual health education and training: For example, consent educators can promote consent as a method of enriching relationships, while also validating the emotional fears that might make one hesitant to practice consent and providing tools to cope with these feelings. Future work should aim to measure these barriers and rewards more precisely; identify individual differences in their endorsement and examine the relation between sexual consent barriers, rewards and behaviour over time.

## Supplemental Material

sj-pdf-1-spr-10.1177_02654075221080744 – Supplemental Material for Perceived barriers and rewards to sexual consent communication: A qualitative analysis Click here for additional data file. Supplemental Material, sj-pdf-1-spr-10.1177_02654075221080744 for Perceived barriers and rewards to sexual consent communication: A qualitative analysis by Jessica Edwards, Uzma S Rehman and E Sandra Byers in Journal of Social and Personal Relationships
