# Supplementary material for: Perceived barriers and rewards to sexual consent communication: A qualitative analysis
Source: J Soc Pers Relat. 2022 Mar 12;39(8):2408–34. doi: 10.1177/02654075221080744 (PMC9294441; doi:10.1177/02654075221080744)
Supplement: sj-pdf-1-spr-10.1177_02654075221080744 – Supplemental Material for Perceived barriers and rewards to sexual consent communication: A qualitative analysis [file sj-pdf-1-spr-10.1177_02654075221080744.pdf]

Supplementary Material

Coding Scheme for Sexual Consent Barriers and Rewards

Note: All examples provided in the coding manual were fictitious and do not reflect actual participant responses.

Sexual Consent Barriers: Thinking about your current and past sexual relationships, what has been the most negative and/or difficult aspect of sexual consent communication?

**Theme**	**Definition**	**Example**
(1) Negative emotions (self)<br>*Note:* do not include "awkwardness" as an emotion	references to own feelings of anxiety, shame, stress, guilt, discomfort, disappointment, embarrassment etc.	"I grew up very conservative, so talking about this openly makes me feel kind of guilty."
(2) Negative emotions (partner)<br>*Note:* do not include "awkwardness" as an emotion	references to partner reacting negatively (e.g., anger, anxiety, shame, disappointment) or becoming offended; not wanting to hurt partner's feelings	"I feel like if you insist on direct communication, there's a chance your partner might think you're accusing them of being untrustworthy"
(3) Inexperience	consent is difficult because the participant or their partner lacks experience with this type of communication<br><br>Note: if it is not clear whether the	"I don't have a ton of experience with this so sometimes I feel kind of out of my depth."

	participant is referring to inexperience with sex or inexperience with consent communication, code for this theme. If the participant is specifically talking about inexperience with sex, do not code.	
(4) Lack of communication skill	consent is difficult because the participant or their partner does not know how to effectively communicate about consent<br>*Note:* this is distinct from not being able to understand the partner (Code #7)	"I never know what to say in these types of conversations."
(5) Initiation	difficult to initiate consent communication; initial communication is more difficult than later communication	"Finding the right moment to talk about it"
(6) Awkwardness	consent feels awkward or unnatural/forced	"If it's too scripted or rigid it can feel really awkward."
(7) Lack of clarity/understanding partner	ambiguity or misunderstandings with regards to consent cues, boundaries, stopping sexual	"Sometimes it's hard to figure out what your partner really wants."

	interaction etc.	
(8) Disrespect or violation of boundaries	partner *deliberately* ignores stated boundaries; violence, coercion, or force	"Well when I told my last partner no he kept whining and trying to convince me, and that was super annoying."
(9) Partner responsiveness	partner unwilling/unable to engage with conversations about consent	"My partner just shuts down whenever I bring it up."
(10) Sexually rejecting/refusing partner	difficulty rejecting the partner<br><br>(include singular instances where one partner wants sex and the other does not)	"It's hard to tell my partner that I don't want to have sex, because I don't want them to feel undesirable"
(11) Being sexually rejected/refused by partner	fear or concern that partner will reject them sexually/not want to have sex; or a negative experience relating to being rejected by the partner<br><br>(include singular instances where one partner wants sex and the other does not)	"When my partner tells me no. It can be a bit disappointing at the time."

(12) Disagreements	general sexual incompatibilities; disagreement about acceptable forms of consent, boundaries, etc. (do not include singular instances where one partner is "in the mood" and the other is not; this would be rejection)	"Sometimes you have this conversation and you find out the other person doesn't care as much as you do"
(13) Relationship impact	consent communication may result in relationship termination, conflict, communication difficulties, decreased satisfaction or trust, etc.	"I don't want to fight about it when I don't want to have sex. It makes everything feel tense."
(14) Impact on sexual interactions/relationship<br><br>*Note:* do not include sexual rejection.	reference to consent "ruining the mood," etc.; makes sex less spontaneous/exciting	"Talking about it makes the sex less exciting."
(15) Context	consent is more difficult depending on external or relational context (e.g., intoxication, early in	"When you or the other person is drunk it can be hard to read signals or even communicate

	relationship, power imbalance)	properly.”
(16) Consent is unnecessary	participant clearly states that they do not value consent	“I don’t think we need to be so strict about consent. People know how to communicate and it really ruins sex when you have to spell it out.”
(17) Other reason not listed	participant identifies another challenge that is not listed here	
(18) None identified – no negative experience	participant clearly states that there have been no negatives in their experience	“Nothing I can think of, my experiences have been pretty good.”
(19) None identified – not enough experience	participant clearly states that they do not have enough experience to identify negative aspects of consent	“I don’t really know, I haven’t had this type of conversation before”
(20) None identified – other	participant does not identify any negatives for a reason not listed here	

Sexual Consent Rewards: Thinking about your current and past sexual relationships, what has been the most positive and/or rewarding aspect of sexual consent communication?

**Theme**	**Description**	**Example**
(1) Relational/ emotional quality	consent enhances relationship quality (e.g., trust, closeness, satisfaction,	“Practicing consent helps you learn to trust each other and feel

	openness, connection)	more connected over time."
(2) Sexual quality	enhances sexual relationship quality (including satisfaction, arousal, intensity, frequency, and safety/health *within the sexual relationship*); being able to experiment/explore or comfortably express sexuality; "consent is sexy"	"Being open with each other makes sex more satisfying. You don't have to wait for them to figure out what you want."
(3) Sexual access	facilitates chance of sexual activity; when partner agrees to have sex/gives consent	"When it leads to sex"
(4) Easy to communicate	consent communication is easy, natural	"Sometimes you just have a good conversation that just flows naturally"
(5) Clarity of expectations	eliminates uncertainty, need for guesswork; prevents misunderstandings	"Confirming that we are both on the same page is really nice"
(6) Openness of communication	makes future consent easier or sets a norm for communication in the relationship	"Once you have the first conversation, talking about it later becomes much less scary."
(7) Streamlined communication	consent becomes more personalized or changes over time (i.e., as you get to know the partner)	"The best thing is how you learn the style of consent that your partner has and over time you can

		drop the formalities and be more natural"
(8) Knowledge of partner	consent communication provides opportunity to learn more about partner	"Learning what they like or don't like is really important"
(9) Safety, respect for boundaries	consent ensures one or both partners' safety, lack of coercion, and/or that boundaries are not violated (incl. safer sex/contraception discussions)	"I can't have sex unless I know the other person can be trusted to listen to me and respect my limits. Consent is a good indicator of that."
(10) Legal and social protection	consent protects parties from lawsuits, false accusations, rumours/reputation damage	"I think it's important to have really clear consent to prevent being accused of something like rape."
(11) Other reason not listed	participant identifies another benefit that is not listed here	
(12) None identified -consent is unnecessary	participant clearly states that they do not value consent and cannot provide a positive	"This isn't necessary so I don't really see a benefit."
(13) None identified – no positive experience	participant clearly states that there have been no positives in their experience	"Honestly, I haven't really had good conversations about consent before. They usually end badly"
(14) None identified	participant clearly states that they do	"I've never really talked about it

– not enough experience	not have enough experience to identify positive aspects of consent	so I wouldn't know."
(15) None identified – other	participant does not identify any positives for a reason not listed here	
